# CSF and Blood Oxytocin Concentration Changes following Intranasal Delivery in Macaque

**DOI:** 10.1371/journal.pone.0103677

**Published:** 2014-08-18

**Authors:** Olga Dal Monte, Pamela L. Noble, Janita Turchi, Alex Cummins, Bruno B. Averbeck

**Affiliations:** 1 Laboratory of Neuropsychology, National Institute of Mental Health, National Institutes of Health, Bethesda, MD, United States of America; 2 Department of Neuropsychology, University of Turin, Turin, Italy; University of Regensburg, Germany

## Abstract

Oxytocin (OT) in the central nervous system (CNS) influences social cognition and behavior, making it a candidate for treating clinical disorders such as schizophrenia and autism. Intranasal administration has been proposed as a possible route of delivery to the CNS for molecules like OT. While intranasal administration of OT influences social cognition and behavior, it is not well established whether this is an effective means for delivering OT to CNS targets. We administered OT or its vehicle (saline) to 15 primates (*Macaca mulatta*), using either intranasal spray or a nebulizer, and measured OT concentration changes in the cerebral spinal fluid (CSF) and in blood. All subjects received both delivery methods and both drug conditions. Baseline samples of blood and CSF were taken immediately before drug administration. Blood was collected every 10 minutes after administration for 40 minutes and CSF was collected once post-delivery, at the 40 minutes time point. We found that intranasal administration of exogenous OT increased concentrations in both CSF and plasma compared to saline. Both delivery methods resulted in similar elevations of OT concentration in CSF, while the changes in plasma OT concentration were greater after nasal spray compared to nebulizer. In conclusion our study provides evidence that both nebulizer and nasal spray OT administration can elevate CSF OT levels.

## Introduction

Oxytocin (OT) is a nonapeptide characterized by a six amino acid ring structure with a three amino acid tail. This molecule is too large to cross the blood–brain barrier (BBB) [1], [2]. The delivery of drugs to the central nervous system (CNS) has been a challenging field of research for several decades, and considerable efforts have been made to enhance the delivery of therapeutic molecules across the vascular barriers of the CNS in an effective and non-invasive manner. A growing literature has suggested that intranasal delivery can be an effective alternative to invasive delivery methods for some classes of these large molecules, including OT [3], [4], [5], [6], as nasal delivery effectively bypasses the BBB.

Studies have shown that intranasal administration of OT in humans influences social information processing, with effects at both behavioral and neural levels [7]. OT has been reported to affect attachment [8], social exploration and memory [9], [10], aggression and anxiety [11], [12], emotion biases [13], and trust [7]. From neuroimaging studies, OT has been shown to modulate amygdala activity in response to threatening social stimuli [14], pain [15], and emotional faces [16].

Although many studies have shown that endogenous OT concentrations in cerebral spinal fluid (CSF) and blood can be measured reliably in humans [17], [18], [19], [20], [21], non-human primates [22], [23] and rodents [24], it is only recently that studies have measured concentration changes in CSF OT levels after intranasal administration [25], [26], [27]. Striepens and colleagues [26] have shown that both CSF and blood OT concentrations increased after OT intranasal administration in human subjects. Although the dose of 24IU resulted in modest increases after 75 minutes, and the sample of subjects used was rather small because of the invasive nature of CSF collection, this study was important as it was the first evidence of OT entering the CNS in humans following nasal delivery. Neumann and colleagues [28] measured changes in central and peripheral OT using micro-dialysis in the amygdala and hippocampus, and blood samples in rats and mice. A peak central increase was found between 30 and 60 minutes after dose delivery. Chang et al. [25] found, with two rhesus macaques, that nasal administration of OT using a nebulizer increases CSF concentrations 30 minutes after delivery; however, no plasma samples were collected in that study. A recent study that evaluated the efficacy of several OT administration routes for elevating central and peripheral OT concentration in monkeys found that only administration of OT with a nebulizer, but not with a nasal spray, significantly increased concentration of OT in CSF [27].

In the present study we build on these findings in several ways using primates. First, we used a larger number of subjects, which is necessary to validate human and non-human findings [25], [26], [27]. Second, because the delivery method most commonly used in human research is a nasal spray, we carried out a direct comparison between nasal spray and nebulizer delivery within the same group of subjects. To address these questions, we administered OT or saline to macaques using either an intranasal spray or a nebulizer, and measured OT concentration changes in the CSF as well as in blood. The CSF sampling was done by cervical puncture at the level of the cisterna magna, it being more proximal to CSF production sites than a lumbar puncture. Each subject received both delivery methods and both drug conditions on separate sessions. This design enabled us to evaluate the efficacy of the intranasal route as well as determine the optimal method for producing consistent OT increases in the CNS.

## Methods and Materials

### Ethics statement

This study was carried out in strict accordance with the recommendations of the National Institutes of Health Guide for the Care and Use of Laboratory Animals. The protocol (LN-23) was approved by the Animal Care and Use Committee of the National Institute of Mental Health. Procedures adhered to applicable United States federal and local laws, regulations and standards, including the Animal Welfare Act (AWA 1990), Regulations (PL 89-544; USDA 1985), and Public Health Service (PHS) Policy (PHS 2002). All experimental procedures were performed under anesthesia, and all efforts were made to minimize suffering. No animals were sacrificed for the purpose of this study.

### Subjects and Procedure

Fifteen male adult rhesus monkeys (*Macaca mulatta*) (6–10 years old, 8–12 kg) participated in this study. All animals were acquired from primate breeding facilities in the United States where they had social-group histories as well as group-housing experience until their transfer to NIH for quarantine. After that, they were pair-housed in a rhesus monkey colony with tactile, auditory, and visual contact with one another. They were tested for tuberculosis and Herpes B twice per year by veterinary staff, and received regular dental checks. Each animal was housed in a standard adult macaque laboratory cage (66 cm width×65 cm length×85 cm height). The colony rooms accommodate 24 rhesus monkeys, and all the primates that served as subjects in this study have been housed at NIH at least 3 to 4 years prior to this experiment. The housing room was maintained on a 12-hour light/dark cycle. Environmental enrichment included 2 toys, most often a chew toy inside the cage, a foraging tube with a variety of food items, and forage board outside the cage that could be readily reached through the cage's bars. All animals were maintained on a diet of fresh fruit and vegetables, and fed 12 monkey chow biscuits (Lab Diet #5038, PMI Nutrition International Inc., Brentwood, MO), twice each day at 7 a.m. and 2 p.m. Animals had unlimited access to water through a bottle attached to the outside of the cage. Healthy body weight was monitored through weekly weight checks.

In this experiment we administered 48IU OT (Sigma) intranasally using an intranasal spray, or in separate sessions a nebulizer, to the 15 male rhesus macaques. This dose has been previously used in primate studies [29]. The same procedure for all animals was repeated with saline as a control measure, thus each subject experienced 4 separate testing sessions separated by at least two weeks. Subjects were male monkeys between 6 and 10 years of age, as it has been shown that CSF OT levels change across lifespan increasing significantly with age [22], and gender [30]. In addition, experimental sessions were conducted at the same time of day to control for diurnal fluctuations [17], [24], [31], [32]. Finally, we used a within-subject design so that each animal would experience both drug conditions and both delivery methods, with a balanced order of administration across animals.

For the intranasal spray condition, atomizers (Intranasal Mucosal Atomization Device, Wolfe Tory Medical, Salt Lake City, UT) were attached to syringes. Anesthetized animals were placed in a supine position with their head tilted back approximately 45 degrees with the chin up so the spray could better reach the epithelium. During each puff in one nostril, the other nostril and the mouth were held closed to allow the solution to reach the respiratory and olfactory epithelia as was done in a previous animal study [33]. A total of 0.8 milliliter (mL) of solution was administered in 4 doses of 200 microliters (µL), alternating nostrils between doses. Administration occurred over 60–90 seconds. For the nebulizer (PARI Baby Nebulizer, PARI Respiratory Equipment, Midlothian, VA) condition the OT solution was diluted to a volume of 2 mL using sterile saline, and was administered over the course of 5–8 minutes. Animals were in the supine position, a cone-shaped facemask designed for canines was fitted entirely over the muzzle area, and the subject breathed freely through the nose for the duration of the dose administration. For both conditions the timing of subsequent sampling was based on the end of the dosing procedure.

On the day of the experiment, before OT or saline intranasal administration, each monkey received an intramuscular dose of ketamine (10 mg/kg) and dexdomitor (0.01 mg/kg) in his home cage and upon sedation was immediately transferred to an adjacent treatment room for blood and CSF fluid collection. Glycopyrrolate (0.01 mg/kg, i.m.) was also administered to reduce brachial secretions and maintain adequate heart rate, which was monitored throughout the procedure with a PulseOximeter. Animals were closely monitored prior to, during, and after anesthesia until they could safely sit upright on their own. Post-procedure analgesics were administered based on consultation with the attending or central facility veterinarian. The regimen most commonly used was ketoprofen (2 mg/kg). Blood samples (2 mL) were drawn from the femoral vein, and CSF (1.5 mL) was obtained from the cisterna magna, as was done in the Chang et al study [25], using a sterile single-use needle. Samples from CSF were taken immediately prior to drug administration and 40 minutes after administration. Blood samples were also taken prior to drug administration and every 10 minutes thereafter, for 40 minutes. For concurrent sample times (baseline and 40 minute time points), blood sample collection was initiated immediately following CSF collection. CSF collection typically took less than 60 seconds. Potential blood contamination of CSF samples was minimized in that a technician with expertise in that procedure collected the samples. All samples were centrifuged prior to assay. Following collection, blood tubes were stored on ice and then centrifuged at 4°C. The resulting plasma aliquots were stored at −80°C. CSF samples were immediately frozen on crushed dry ice and subsequently stored at −80°C. We were able to obtain sufficient CSF and plasma samples from 15 monkeys for all-time points under all drug conditions and delivery methods.

### Measurement of OT concentration in samples

Previous research has used un-extracted CSF samples to estimate OT concentration in rodents [35], in primates [22], , in human neonates [36], and vasopressin concentration in humans [3]. We also used this approach in the present study. All samples were assayed for OT by enzyme-linked immunosorbent assay (*ELISA*) using a commercially available kit (Enzo Life Sciences, Farmingdale, NY). The kits have a sensitivity of 11.7 pg/mL with less than 0.2% cross-reactivity with vasopressin. Instructions supplied with the OT ELISA kit were followed without modification, and CSF samples were directly assayed while plasma was extracted prior to testing [27], [37]. Plasma was extracted using acetone and ether, and dried down using a Savant speedvac as outlined by Enzo Life Sciences technical support. Plasma samples were then rehydrated in assay buffer prior to testing. We examined serial dilutions and only extracted plasma produced linear concentration estimates (preliminary data).

### Statistical Analyses

For analysis of CSF OT concentration we used a 2×2×2 repeated measures ANOVA with drug (OT and saline), time (pre and post administration), and delivery method (intranasal spray and nebulizer) as within-subject factors. For analysis of plasma OT concentration at pre and 40 minute time points, as for CSF, we used a 2×2×2 repeated measures ANOVA with drug (OT and saline), time (pre and post administration), and delivery method (intranasal spray and nebulizer) as within-subject factors. Where appropriate, *post hoc* t-test comparisons were performed to examine interaction effects with two-tailed independent t-tests. All p-values were Bonferroni corrected for the number of comparisons. P-values that exceeded 1 after Bonferroni correction are reported as 1. In the second analysis we tested the time course of OT concentration in plasma and we used a 5×2×2 repeated measures ANOVA with time (1 pre and 4 post time points), drug (OT and saline), and delivery method (intranasal spray and nebulizer) as within-subject factors. Where appropriate, *post hoc* t-test comparisons were performed (the p-value was Bonferroni corrected), to examine interaction effects.

## Results

### CSF OT concentration changes

**Figure 1:**
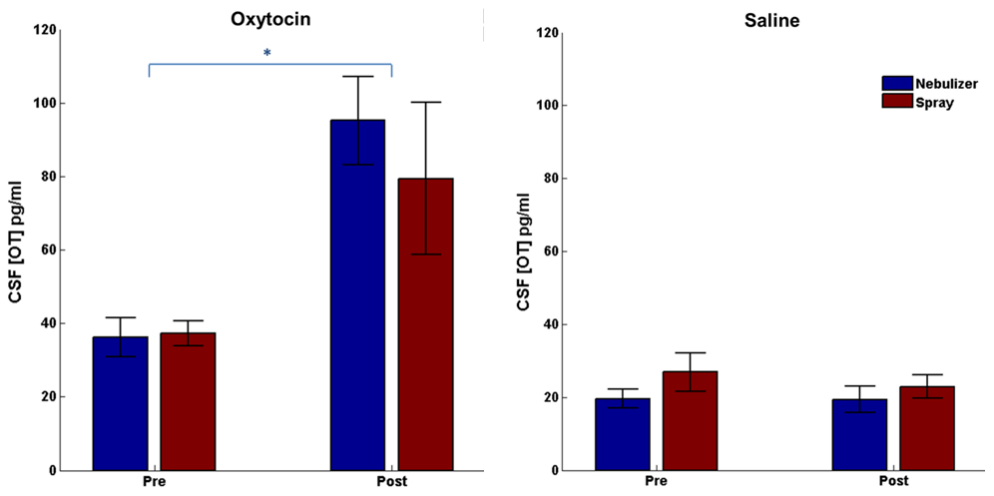
Cerebral spinal fluid (CSF) Oxytocin (OT) concentration. Values shown are the mean ± SEM (n = 15). **A.** OT concentration in CSF before and 40 minutes after intranasal OT administration, *p<.05. **B.** OT concentration in CSF before and 40 minutes after intranasal saline administration.

Both the intranasal spray and nebulizer administration of OT resulted in an elevation of OT concentration in CSF (Figure 1, drug by time; F(1,14) = 19; *p* = .001). There was no difference between the spray and nebulizer delivery methods (method; F(1,14) = .048; *p* = .831). Nor did the changes in CSF OT concentration between pre and post sample times differ between the two delivery methods (method by time; F(1,14) = .78; *p* = .391). There was also no difference between methods across drug conditions (method by drug; F(1,14) = 1.8, *p* = .201). Because there was no main effect of delivery method, we collapsed them together to confirm the significant increase of OT in CSF after OT administration compared to saline, and analyzed differences between pre- and post- OT and saline administration. We found that CSF OT levels were significantly elevated 40 minutes after delivery relative to baseline when OT was administered (t(14) = 4.4, *p* = .004) but not when saline was administered (t(14) = .71, *p* = 1). There was no CSF OT concentration difference at baseline between the OT and saline conditions (t(14) = 2.6, *p* = .092); but there was a significant difference in CSF concentration between OT and saline after administration (t(14) = 5.8, *p*<.001). Thus we saw statistically significant increases of OT levels in CSF after OT administration but not after saline administration with both delivery methods. Although there was no significant difference between administration methods for OT, we ran an additional analysis to investigate if there were differences in the statistical reliability of the delivery methods. We found that OT levels increased more robustly 40 min after nebulizer administration compared to baseline (t(14) = 4.8, *p*<.001) than after nasal spray (t(14) = 2.25, *p* = .041).

### Plasma OT concentration changes

**Figure 2:**
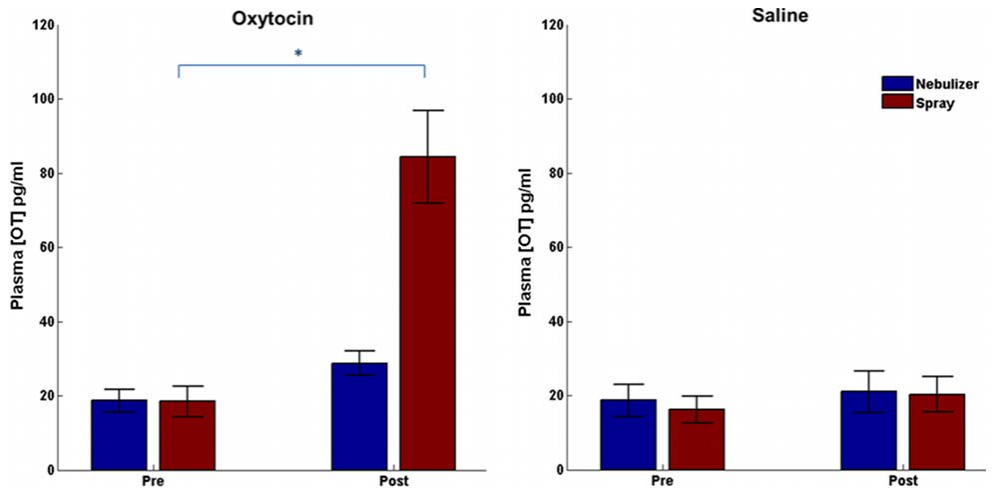
Plasma Oxytocin (OT) concentration. Values shown are the mean ± SEM (n = 15). **A.** OT concentration in plasma before and 40 minutes after OT intranasal administration, *p<.05. **B.** OT concentration in plasma before and 40 minutes after intranasal saline administration.

Concurrent measurement of OT concentration in plasma (Figure 2) showed a significant increase in OT at 40 minutes relative to baseline (drug by time, F(1,14) = 36.38; *p*<.001). Furthermore, there were differences in methods across time. OT levels after OT administration increased more from baseline for intranasal spray than for nebulizer delivery (method by drug by time, F(1,14) = 11.85, *p* = .004). We next carried out post-hoc analyses comparing the two delivery methods, time, and drugs administered. While there was no significant difference between the two methods in the saline condition at baseline (t(14) = 0.41, *p* = 1) or after 40 minutes (t(14) = 0.21, *p* = 1), we found that plasma OT levels were significantly elevated 40 minutes after OT nasal spray delivery relative to nebulizer (t(14) = 3.92, *p* = .008). Plasma OT levels also did not differ between the two delivery methods at baseline (t(14) = 0.05, *p* = 1) before OT administration. Furthermore, when we investigated OT concentration changes within delivery methods, we found that OT was significantly increased 40 minutes after nasal spray administration compared to baseline (t(14) = 5.2, *p*<.001), but not after nebulizer (t(14) = 2.19, *p* = .09).

### Time course of OT plasma concentration changes

**Figure 3:**
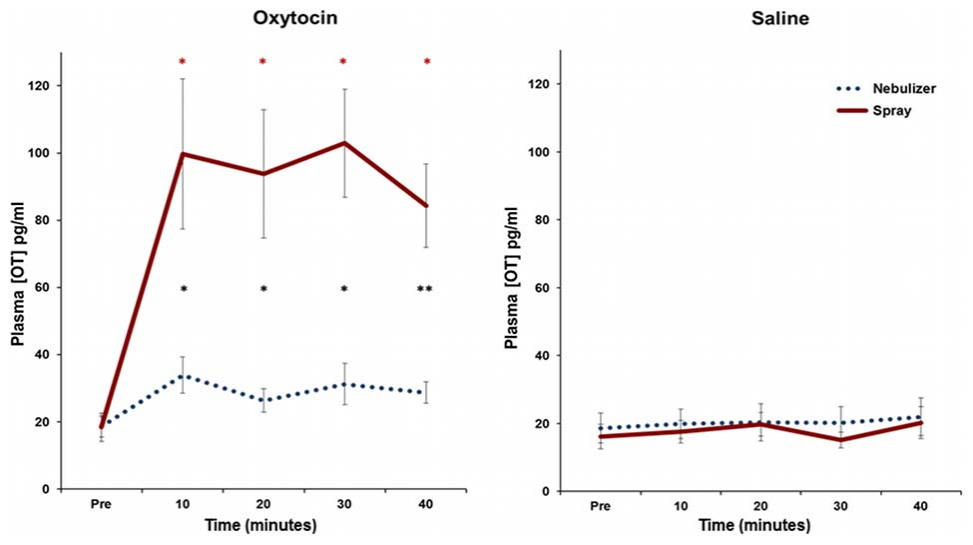
Plasma Oxytocin (OT) concentration. Values shown are the mean ± SEM (n = 15). **A.** OT concentration in plasma at each time point before and after OT administration. Note that asterisks in black show significant differences between the two delivery methods. Red asterisk indicates significant elevation of OT concentration for each time point relative to baseline for the intranasal spray condition; *p<.05 and **p<.001. **B.** OT concentration in plasma at each time point before and after saline administration.

When the time course of plasma OT was analyzed (Figure 3), we found interactions between drug and method (F(1,14) = 15.6; *p* = .001), drug and time (F(4,11) = 10.1; *p*<.001), and time and method (F(4,11) = 6.8; *p*<.001). In addition, there were differences in the two delivery methods over time (drug by time by method F(4, 11) = 6.9, *p* = .023). We next carried out post-hoc analyses at each time point, comparing methods. While there was no significant difference at baseline (t(14) = 0.05, *p* = 1), we found effects of method for each time point after OT delivery: at 10 minutes (t(14) = 3.22, *p* = .03), 20 minutes (t(14) = 3.59, *p* = .015), 30 minutes (t(14) = 4.64, *p* = .005), and 40 minutes (t(14) = 3.92, *p* = .001). Additionally, we performed post-hoc analyses examining the effect of drug at each time point for each delivery method. For nebulizer administration, OT levels were not significantly elevated relative to baseline after 10 minutes (t(14) = 2.56, *p* = .092), 20 minutes (t(14) = 1.59, *p* = .54), 30 minutes (t(14) = 1.72, *p* = .43), or 40 minutes (t(14) = 2.198, *p* = .216) following OT delivery. For nasal spray OT administration, OT levels were significantly elevated at all-time points relative to baseline: at 10 minutes (t(14) = 3.82, *p* = .008), 20 minutes (t(14) = 4.04, *p* = .004), 30 minutes (t(14) = 5.25, *p* = .004), and 40 minutes (t(14) = 5.21, *p* = .004) after OT administration. Following nebulizer delivery the OT concentrations returned to baseline at the end of 40 minutes; in contrast, OT levels after intranasal spray did not return to baseline before the end of the sampling period.

## Discussion

Our present findings, consistent with previous studies, show that intranasal administration of exogenous OT increases the concentration of this peptide both in CSF and plasma compared to saline. Both the intranasal spray and nebulizer administration methods resulted in elevations of OT concentration in CSF. Concurrent measurement of OT concentration in plasma showed an increase in plasma OT levels after OT administration but not after saline; however, the changes in plasma OT concentration between pre- and post-sample times differed significantly between the two delivery methods, with a greater elevation of OT after nasal spray compared to nebulizer. When we investigated the time course of OT concentration change in plasma, we found that following nebulizer delivery the OT concentration returned to baseline at the end of 40 minutes, while OT levels after intranasal spray did not return to baseline before the end of the sampling period.

Consistent with recent findings in humans [26] non-human primates [25], [27] and rodents [28], we found that intranasal administration of OT increased OT levels in CSF compared to intranasal administration of saline. Striepens et al., [26] is the first study with human participants to test CSF OT levels after OT intranasal administration. Using a between-subject design they found an OT concentration increase in CSF after 75 minutes in 3 subjects using a 24IU dose of OT intranasal administration, compared to 1 subject that underwent saline administration. This result is in line with previous human [3] and animal studies [38], [39] that have shown that some peptides (insulin, vasopressin, nerve growth factor) increase in CSF after intranasal administration. Born reported significant increases in CSF concentration following administration of vasopressin between 40 and 80 minutes [3]. The first study in non-human primates to investigate concentration changes in CSF OT levels [25] reported that following nebulizer administration of 25IU of OT, CSF OT concentrations increased after 35 minutes from 20 to 50 pg/ml in 2 rhesus monkeys. The second primate study found that CSF OT concentration increased after 60 and 120 minutes from 0.3 to 0.7 pg/ml in 4 monkeys [27]. Neumann and colleagues [28] measured changes in central and peripheral OT using a pipette to deliver exogenous OT to the rhinarium in 11 anesthetized rats and 12 mice and used microdialyis to sample from the amygdala and dorsal hippocampus. They found a peak OT increase between 30 and 60 minutes after OT administration.

Our findings add to these human [26] and primate studies [25], [27] by using a sample size of 15 male monkeys, and a within-subject design where each subject received both delivery methods and both drug conditions over 4 separate sessions, with the order of condition and delivery method randomized. However, there is some discrepancy between the time courses of OT changes in CSF across studies. While our results showed an increase 40 minutes after intranasal administration, which generally agrees with results in the Chang et al [25] and Neumann and colleagues studies [28], the time courses found in Modi et al [27] and the human study [26] differ in that the increases were not detected until 60 minutes and 75 minutes after dose administration, respectively. We did not assay these time points in the current study, so a direct comparison of level of elevation is not possible, but the differences could be explained by the fact that in our study the CSF sampling was done through cervical puncture at the cisterna magna, rather than a lumbar puncture that is much more distal to production sites. Distal sampling requires more time for changes to be detected, and also allows more opportunity for the OT to diffuse and be metabolized before reaching the lumbar area.

The present study sought to validate the human and primate results by using more subjects, and to compare the efficacy of two commonly used delivery methods: nasal spray in human studies [40], and nebulizer in primate studies [25], [27], [29]. Our current findings demonstrate that both delivery methods increased OT levels in CSF compared to saline, thus supporting the recent results in humans [26] and animals [25], [28], and indicating that both delivery methods are viable. However, contrasting findings were reported by Modi and colleagues [27] where the nebulizer, but not the nasal spray, increased CSF OT concentration after administration. This discrepancy could be explained by the number of subjects, the within- versus between-subject design, the radioimmunoassay (RIA) instead of ELISA used to analyze the CSF samples, different time points measured, different doses, different CSF sapling sites, and different OT concentrations detected at baseline (i.e. they were 10-fold smaller than our data and those reported in other studies [20], [23], [25], [36]).

Some have queried whether the elevated OT in the CNS is actually due to the intranasally administered OT crossing into the CNS [41], or if it instead triggers an increase in endogenous OT production without crossing the BBB [42]. While this question is not clinically relevant, as therapeutic elevations in CSF OT due to peripheral administration could be beneficial regardless of route of entry, learning more about the mechanism would be valuable for pharmaceutical development. It may also be possible that CNS OT is elevated via indirect effects of peripheral elevation; further studies tracking OT diffusion are needed to answer this question.

We have shown that both the nebulizer and intranasal spray delivery methods resulted in central increases of OT. The nebulizer delivery was, however, more robust across subjects, but the changes in plasma OT concentration between pre- and post-sample times differed significantly between the two delivery methods. Consistent with previous human studies that have shown increase in OT plasma concentration 30–40 minutes after intranasal administration [43], [44] we found a significant elevation of OT levels after nasal spray, compared to saline. This may be due to the formation of more or larger droplets of fluid using the spray, which would rest directly on the nasal mucosa allowing for greater opportunity for vascular absorption compared to the nebulizer, which forms a much finer mist, allowing more of the substance to be breathed in deeper towards the epithelium. Our results are in agreement with a recent study in non-human primates where only the nasal spray OT administration resulted in peripheral increases of OT, but not the nebulizer [27]. Thus, while both methods resulted in the same CNS penetration, the differences in peripheral action must be considered when choosing between them.

Furthermore, the time course of OT plasma concentration changes was found to differ between delivery methods, in that following nebulizer delivery the OT concentrations had a smaller initial increase at 10 minutes and had returned to baseline levels at the end of 40 minutes, while intranasal spray concentrations had a much sharper increase at 10 minutes [26], and did not return to baseline before the end of the sampling period. This is consistent with previous humans studies reporting that both vasopressin [3] and OT concentration was elevated compared to baseline 80–90 minutes after intranasal administration [43], and with a non-human primate study where plasma OT concentration was elevated 120 minutes after intranasal spray compared to baseline [27]. As discussed above, the use of the nasal spray may cause a greater increase in plasma OT because of the larger droplets that form on the nasal mucosa, allowing for more vascular absorption compared to the nebulizer. However, because our animals were anesthetized and in a supine position, this effect may have been even greater than in awake, erect animals, as the fluid would not run out of the nasal cavity as in an upright position, which may lead to the higher blood OT concentration.

Whether plasma OT level is indicative of central release patterns and activities within the brain and therefore associated with social behaviors remains unknown. In our data we could not investigate if there was a significant correlation between peripheral and central OT levels, because the CSF and plasma samples were not treated in the same way. We did not use extraction on the CSF samples, but we did use extraction on the plasma samples. Many studies have tried to answer this question, but most of those also assayed peripheral and central OT samples differently, making interpretation of the data difficult [45]. There are two main problems reported in the literature related to OT analysis due to methodological differences between studies. The first concerns how much sample preparation influences quantification of neuropeptides in an assay; the second is in regards to how much OT the most commonly used assays are actually measuring. The measurement of peripheral OT started with the development of RIA methods [46]. Almost 30 years later researchers began developing ELISA to avoid the use of radioactive materials. In the ELISA, there were divergent results in un-extracted compared to extracted samples: un-extracted plasma yielded values 100-fold greater than the same sample after extraction [47]. For example, humans have been reported to have basal plasma OT concentrations ranging between 0.1–23 pg/ml in extracted plasma and 99–405 pg/ml in raw plasma. The effect of extraction was also evident in samples measured by RIA, where un-extracted plasma gave values that were at least 10-fold higher than the extracted sample (reviewed in Szeto et al [48]). Although it is currently unknown whether extraction prevents cross-reactivity, or whether it eliminates the effects of binding proteins or other substances that interfere with the assay, OT-free plasma (for instance, obtained from OT knockout mice) could be used as a negative control to evaluate the issue further.

While discrepancies between results obtained with and without extraction using different assays have started to be addressed in plasma samples [47], the issue has not received the same attention for CSF samples. Most of the studies that have assayed OT in CSF used un-extracted samples [22], [23], [34], [35], [36]; only a few studies have used extracted CSF samples with RIA [20], [26], [27], [28], and only one used extracted samples with ELISA [24]. However, OT levels do not seem to differ as much between extracted and un-extracted CSF samples compared to plasma. Two studies with rhesus monkeys analyzed un-extracted CSF OT samples and reported baseline levels between 20 and 30 pg/ml [23], [25]. Human studies reported baseline CSF OT levels between 10 and 34 pg/ml without extraction [36] and around 20 pg/ml with extraction [20], [26]. Only one study reported much lower baseline CSF OT levels in monkeys that range between 0.2 and 0.4 pg/ml after sample extraction [27].

Whether peripheral measures of neuropeptides are a reliable estimate of central concentration remains an important research question and more work needs to be done to investigate the validity of measurements of OT in peripheral fluids such as plasma, saliva, and urine as a global biomarker of behaviorally relevant concentrations of central OT. Until assays are strictly validated and standardized to detect bioavailable neuropeptides, interpretation of the data remains difficult. The protocol followed when collecting and analyzing any type of biological samples for OT does have an impact on the results obtained.

## Conclusions

Although the last decade has seen a large number of published findings adding knowledge to this topic, more effort is needed to investigate the mechanisms of intranasal administration of OT and its effect at both the central and peripheral levels. Specifically, future studies should address the pharmacokinetics of this peptide, as it is still not known how much OT reaches target sites in the brain. Second, the time course of exogenous OT should be measured with serial CSF sampling from an implanted brain or cervical port. Third, measurement of the exact amount of exogenous OT that reaches the CNS following intranasal administration must be carried out, as well as testing of different dosages in order to determine the minimum amount required to trigger central effects. Fourth, the validity of peripheral OT measurement as a biomarker for central function is still debated and there is a critical need to establish valid and reliable methods to measure OT in plasma and other biological fluids. Our findings provide evidence that intranasally administered OT does elevate CNS OT levels as well as peripheral levels. Overall, our study supports the hypothesis that delivering OT intranasally, either with a nebulizer or with a nasal spray, which has been used regularly to test social cognition in humans, results in elevations of OT in CSF.
